# Association of child maltreatment subtypes and long-term physical health in a German representative sample

**DOI:** 10.1080/20008198.2018.1510278

**Published:** 2018-09-07

**Authors:** Vera Clemens, Markus Huber-Lang, Paul L. Plener, Elmar Brähler, Rebecca C. Brown, Jörg M. Fegert

**Affiliations:** a Department of Child and Adolescent Psychiatry/Psychotherapy, University of Ulm, Ulm, Germany; b Institute of Clinical and Experimental Trauma Immunology, Ulm University Medical Centre, Ulm, Germany; c Department of Psychosomatic Medicine and Psychotherapy, University Medical Center of Johannes Gutenberg University Mainz, Mainz, Germany

**Keywords:** Child abuse and neglect, child maltreatment, physical health outcomes, obesity, diabetes, cancer, hypertension, chronic obstructive pulmonary disease, myocardial infarction, stroke, Abuso infantil y negligencia, Maltrato infantil, Consecuencias de Salud Física, Obesidad, Diabetes, Cáncer, Hipertensión Arterial, Enfermedad pulmonar obstructiva crónica, Infarto al Miocardio, Apoplejía, 虐待和忽视儿童, 童年虐待, 身体健康结果, 肥胖, 糖尿病, 癌症, 高血压, 慢性阻塞性肺疾病, 心肌梗塞, 中风, • Studies addressing cumulative effects of different child maltreatment subtypes on physical health are sparse, especially those comprising emotional and physical neglect.• Odds for obesity, diabetes, cancer, hypertension, chronic obstructive pulmonary disease, history of myocardial infarction and stroke increased when any kind of child maltreatment was reported.• Growing intensity of each maltreatment subtype was associated with higher rates of all assessed conditions.• Odds for all conditions increased with increasing number of maltreatment subtypes that were experienced.

## Abstract

**Background**: Child maltreatment is a major public problem, associated with enormous consequences on the individual and socioeconomic level. Studies show a clear impact of child maltreatment on long-term physical health. However, there is a lack of analyses comprising a wide variety of subtypes of maltreatment and addressing cumulative effects of different maltreatment subtypes experienced during childhood on physical health.

**Objective**: The objective of this analysis was to assess the association of different subtypes and the intensity of child maltreatment with long-term physical health outcomes.

**Methods**: In a cross-sectional observational approach, a representative sample of the German population (N=2510) was assessed regarding socioeconomic information, their current health status, and their experiences of child maltreatment using the Childhood Trauma Questionnaire (CTQ). Chi^2^-Tests were performed to compare differences of physical health conditions in adulthood in association with child maltreatment and binary regression analyses to assess the relationship of physical health and number of different subtypes of maltreatment experienced during childhood.

**Results**: Odds increased significantly for obesity (1.8), diabetes (1.26), cancer (1.28), hypertension (1.16), chronic obstructive pulmonary disease (1.51), history of myocardial infarction (1.29) and stroke (1.31) with increasingenhancing number of experienced subtypes of child maltreatment. Growing intensity of each subtype of maltreatment was associated with higher rates of all assessed physical health conditions, which could point towards a dose-dependency of the relationship between maltreatment and long-term physical health.

**Conclusions**: Child maltreatment is associated with increased odds for the leading morbidity and mortality causes in Germany. Interventions encompassing secondary and primary preventive strategies are critical to target this major public health problem and its devastating consequences.

## Abstract (es)

**Antecedentes:** El maltrato infantil es un problema público mayor, asociado a enormes consecuencias a nivel individual y socioeconómico. Los estudios muestran un impacto claro del maltrato infantil sobre la salud física a largo plazo. Sin embargo, existe una carencia de análisis que comprendan una amplia variedad de subtipos de maltrato y aborden los efectos acumulativos de diferentes subtipos de maltrato experimentados durante la infancia sobre la salud física.

**Objetivos:** El objetivo de este análisis fue evaluar la asociación de diferentes subtipos y la intensidad del maltrato infantil con consecuencias en la salud física a largo plazo.

**Métodos:** En un enfoque observacional transversal, se evaluó una muestra representativa de la población germana (N=2510) en cuanto a información socioeconómica, su estado de salud actual, y sus experiencias de maltrato infantil usando el Cuestionario de Trauma Infantil (CTQ por sus siglas en Inglés). Se realizaron tests de Chi^2^ para comparar diferencias de condiciones de salud física en la adultez en asociación con maltrato infantil y análisis de regresión binaria para evaluar la relación entre salud física y número de diferentes subtipos de maltrato experimentado durante la infancia.

**Resultados:** Las probabilidades (expresadas en O.R.) aumentaron significativamente para obesidad (1.18), diabetes (1.26), cáncer (1.28), hipertensión arterial (1.16), enfermedad pulmonar obstructiva crónica (1.51), historia de infarto al miocardio (1.29) y apoplejía (1.31) con número mayor de subtipos de maltrato infantil experimentados. La intensidad creciente de cada subtipo de maltrato se asoció con tasas mayores de todas las condiciones de salud física evaluadas, lo que puede apuntar a una dependencia de dosis de la relación entre maltrato y salud física a largo plazo.

**Conclusiones:** El maltrato infantil se asocia con aumento de probabilidades para las causas principales de morbilidad y mortalidad en Alemania. Intervenciones que abarquen estrategias preventivas secundarias y primarias son críticas para impactar este problema de salud publica mayor y sus consecuencias devastadoras.

## Abstract (zh)

**背景：**儿童虐待是一个重大公共问题，给个人和社会经济带来严重后果。研究表明童年虐待对长期身体健康有明显影响。然而，还缺乏相关研究对多种虐待亚型进行分析，并关注儿童期所经历的不同虐待亚型对身体健康的累积效应。

**目标：**该分析的目的是评估童年虐待的不同亚型及其强度与长期身体健康状况的关联性。

**方法：**在横断观察研究中，搜集了一个德国人口的代表性样本（N = 2510）的社会经济信息，目前的健康状况，并使用儿童创伤问卷（CTQ）评估其童年虐待的经历。使用卡方检验比较不同成年期身体健康状况与儿童虐待的关系的差异；使用二元回归分析考察身体健康与儿童期不同虐待亚型数量之间的关系。

**结果：**肥胖（1.18），糖尿病（1.26），癌症（1.28），高血压（1.16），慢性阻塞性肺病（1.51），心肌梗死病史（1.29）和中风（1.31）的概率随着童年所经历虐待亚型数量的增加而显著增加。不论哪种虐待亚型，其强度增加与所有被评估的身体健康问题的比率升高相关，这可能说明虐待与长期身体健康之间关系具有‘程度相关性’（dose dependency）。

**结论：**儿童期虐待与德国几大首要致病和致死疾病的发生率增加有关。制定干预措施，设计次要和主要预防方案，对解决这一重大公共卫生问题和减轻其严重后果至关重要。

## 1. Introduction

Child maltreatment is a major public health problem (Norman et al., 2012). Along with very high prevalence rates – one-third of adults report that they experienced some form of maltreatment during childhood (Witt, Brown, Plener, Brahler, & Fegert, 2017) – the consequences of child maltreatment are enormous. Psychosocial impairment and economic impact, a significant reduction in quality of life and a strikingly increased morbidity, including both mental and somatic health problems, have been reported (Norman et al., 2012). Child maltreatment leads to a significant reduction in lifespan of up to 20 years (Brown et al., 2009). Beside its devastating consequences on the life of each individual victim, child maltreatment results in an enormous economic burden, with annual costs between 11 and 30 billion euro in Germany alone (Habetha, Bleich, Weidenhammer, & Fegert, 2012).

Child maltreatment, defined as ‘any act or series of acts of commission or omission by a parent or other caregiver that results in harm, potential for harm, or threat of harm to a child’ (Leeb, Paulozzi, Melanson, Simon, & Arias, 2008), can be divided into five subtypes: emotional, physical and sexual abuse, and emotional and physical neglect. A recent epidemiological study from a German sample reports at least moderate experience of emotional abuse in 6.6%, physical abuse in 6.7%, sexual abuse in 7.6%, and emotional and physical neglect in 13.3% and 22.5%, respectively (Witt et al., 2017).

In the first large epidemiological study in the USA assessing the impact of maltreatment during childhood on physical health in adulthood, Felitti et al. (1998) showed that subjects who had experienced child maltreatment had an increased risk for several diseases which are among the leading causes of death worldwide. Depending on the number of maltreatment experiences encountered, the odds ratio (OR) was reported to be increased by up to 1.6 for severe obesity, 2.2 for ischaemic heart disease, 1.9 for the occurrence of any kind of cancer, 2.4 for stroke, 3.9 for chronic bronchitis or emphysema and 1.6 for diabetes (Felitti et al., 1998). Other extended cross-sectional studies followed, confirming associations of child maltreatment with an enhanced risk for obesity, hypertension, diabetes, emphysema and cardiovascular diseases, including stroke and myocardial infarction (Afifi, Mota, MacMillan, & Sareen, 2013; Draper et al., 2008; L. K. Gilbert et al., 2015).

For individual physical health conditions, longitudinal studies were conducted to assess the impact of child maltreatment on physical health in adulthood. Thus, several longitudinal studies (Johnson, Cohen, Kasen, & Brook, 2002; Noll, Zeller, Trickett, & Putnam, 2007; Thomas, Hypponen, & Power, 2008) and a meta-analysis (Danese & Tan, 2014) pointed towards an increased risk for obesity in adulthood if any childhood trauma had occurred. Furthermore, higher risks for cardiovascular disease (Doom, Mason, Suglia, & Clark, 2017) and hypertension (Suglia, Clark, Boynton-Jarrett, Kressin, & Koenen, 2014) after child maltreatment were reported in longitudinal assessments.

Even though some studies focused on the impact of specific maltreatment subtypes, such as sexual (Maniglio, 2009) or physical abuse (Afifi et al., 2013; Springer, Sheridan, Kuo, & Carnes, 2007) on physical health outcomes, since different methods were used, these results are hardly comparable. Two recent publications from a Canadian survey assessed the impact of physical abuse, sexual abuse and exposure to intimate partner violence during childhood on later development of diabetes and chronic obstructive pulmonary disease (COPD). The data point towards a major influence of sexual abuse on diabetes and COPD compared to the influence of physical abuse and exposure to partner violence. Furthermore, a cumulative effect was found when a combination of two or three maltreatment types was experienced (Shields, Hovdestad, Gilbert, & Tonmyr, 2016; Shields, Hovdestad, Pelletier et al., 2016). This pivotal effect of sexual abuse, even in comparison to other maltreatment types, was also reported regarding the development of hypertension (Suglia et al., 2014).

Taken together, there is striking evidence pointing towards child maltreatment as a severe risk factor for physical health. Nevertheless, although a high intercorrelation between different subtypes of maltreatment, as well as a cumulative effect of different maltreatment subtypes on long-term physical health, is well known (Draper et al., 2008; Felitti et al., 1998; Shields, Hovdestad, Gilbert et al., 2016; Shields, Hovdestad, Pelletier et al., 2016), there is a lack of analyses focusing on cumulative effects of different types of maltreatment by involving the number of subtypes of maltreatment experienced. Moreover, there is a lack of studies analysing the association of single subtypes of maltreatment and physical health. In particular, the relationship between emotional and physical neglect and physical health outcomes has rarely been assessed, even though neglect occurs much more commonly than other forms of maltreatment (Witt et al., 2017). Therefore, in an epidemiological, cross-sectional approach, using data from a representative sample of the German population, we sought to analyse the odds for different physical health conditions depending on the experience of different subtypes of child maltreatment, and, furthermore, conducted a regression analysis assessing the association with the total number of subtypes experienced.

## 2. Methods

Using a random route procedure, a representative sample of the German population was obtained by a demographic consulting company (USUMA, Berlin, Germany). Data collection took place between September and November 2016. The sample was representative of the German population above the age of 14 years with regard to age, gender and geographic region. Households of every third residence in a randomly chosen street were invited to participate in the study. In multi-person households, participants were randomly selected using a Kish selection grid. For inclusion, participants had to be at least 14 years of age and have sufficient German language skills. Of 4902 designated addresses, 2510 households participated in the study. The main reasons for non-participation were failure to contact anyone in the residence after four attempts (14.9%), refusal by the individual who answered the door to have anyone in the household participate in the study (15.3%), failure to contact the randomly selected household member after four attempts (2.3%) and refusal by the selected member to participate (14.7%).

Individuals who agreed to participate were given information about the study and provided informed consent. In the case of minors, participants gave informed assent with informed consent being provided by their caregivers. Participants were told that the study was about psychological health and well-being. Responses were anonymous. In the first step, sociodemographic information was obtained in an interview format by the research staff. Then, the researcher handed out a copy of the questionnaire and a sealable envelope. The researcher either remained nearby in case the participants needed further information or left the household based on the participants’ wishes. Either way, the researcher did not interfere with the filling out of the questionnaire. The completed questionnaires were linked to the respondents’ demographic data, but did not contain their name, address or any other identifying information.

The study was conducted in accordance with the Declaration of Helsinki, and fulfilled the ethical guidelines of the International Code of Marketing and Social Research Practice of the International Chamber of Commerce and of the European Society of Opinion and Marketing Research. The study was approved by the Ethics Committee of the Medical Department of the University of Leipzig.

### 2.1. Measures

The prevalence of five types of child maltreatment was assessed using the 28-item version of the Childhood Trauma Questionnaire (CTQ) (Bernstein et al., 2003; Klinitzke, Romppel, Hauser, Brahler, & Glaesmer, 2012; Wingenfeld et al., 2010). The CTQ is a screening measure for the assessment of child maltreatment. It contains five subscales, each assessed by five items, namely sexual, emotional and physical abuse, and emotional and physical neglect. An additional three items assess whether participants tend to trivialize problematic experiences within their family. Valid and reliable psychometric properties of the German version of the CTQ were demonstrated by Klinitzke et al. (2012), with internal consistencies ranging between 0.62 and 0.96 for all subscales. The intraclass coefficient for an interval of 6 weeks was 0.77 for the overall scale and between 0.58 and 0.81 for the subscales.

Based on norm data by Hauser, Schmutzer, Brahler, and Glaesmer (2011), severity scores for each subscale can be calculated, ranging from ‘none–minimal’ through ‘minimal–moderate’ and ‘moderate–severe’ to ‘severe–extreme’. Dichotomous scores (e.g. experience of emotional neglect: yes/no when used to analyse the number of experienced forms of child maltreatment) were based on scores reaching at least the moderate–severe level.

### 2.2. Participants

Of the *N* = 2510 participants, between 2411 and 2436 participants (depending on their health condition) were included in the sample; the others were excluded owing to missing data on particular health outcomes. Participants were on average 48.4 years old (SD = 18.2) and 53.3% were female. A place of birth outside Germany was reported by 3.2%. The sample was representative of the German population in regard to age and gender. The characteristics of the sample are presented in Table 1.

### 2.3. Statistical analyses

All analyses were conducted using SPSS version 21. Descriptive analyses were performed for prevalence rates. Comparisons were conducted using chi-squared tests.

Binary logistic regression analyses were performed to identify predictors of physical health conditions. Age (in years, continuous variable), gender, smoking, risk of alcohol abuse and educational level (achieved baccalaureate yes/no) were entered in the analyses as covariates.

## 3. Results

A total of *n* = 772 (30.8%) of the participants reported at least one type of maltreatment. In detail, 163 (6.5%) reported emotional abuse, 167 (6.7%) physical abuse, 190 (7.6%) sexual abuse, 332 (13.3%) emotional neglect and 562 (22.5%) physical neglect. A detailed description of child maltreatment in the sample can be seen elsewhere (Witt et al., 2017).

A total of *n* = 416 (16.7%) of the participants reported that they were obese, while *n* = 164 (6.6%) stated that they had diabetes and *n* = 579 (23.3%) hypertension. Furthermore, *n* = 31 (1.2%) of the participants stated that they had COPD, while *n* = 108 (4.4%) reported a history of cancer, *n* = 59 (2.4%) a history of myocardial infarction and *n* = 55 (2.2%) a history of stroke (Table 1).

**Table 1.** Sample characteristics.

Age (years), mean (SD)	48.4 (18.2)
Gender	
Female	1339 (53.3)
Male	1171 (46.7)
Risk of alcohol abuse	298 (11.9)
Smoking	841 (33.5)
High educational level (baccalaureate)	543 (21.6)
Child maltreatment (CTQ)	
Emotional abuse	163 (6.5)
Physical abuse	167 (6.7)
Sexual abuse	190 (7.6)
Emotional neglect	332 (13.3)
Physical neglect	562 (22.5)
Ever experienced any form of child maltreatment	772 (30.8)
Obesity	416 (16.7)
Diabetes	164 (6.6)
Cancer	108 (4.4)
Hypertension	579 (23.3)
Myocardial infarction	59 (2.4)
COPD	31 (1.2)
Stroke	55 (2.2)

Data are presented as number of subjects (%) unless otherwise indicated.

COPD, chronic obstructive pulmonary disease.

### 3.1. Differences in health conditions in adulthood in association with different subtypes and intensity of child maltreatment

**Figure 1.**
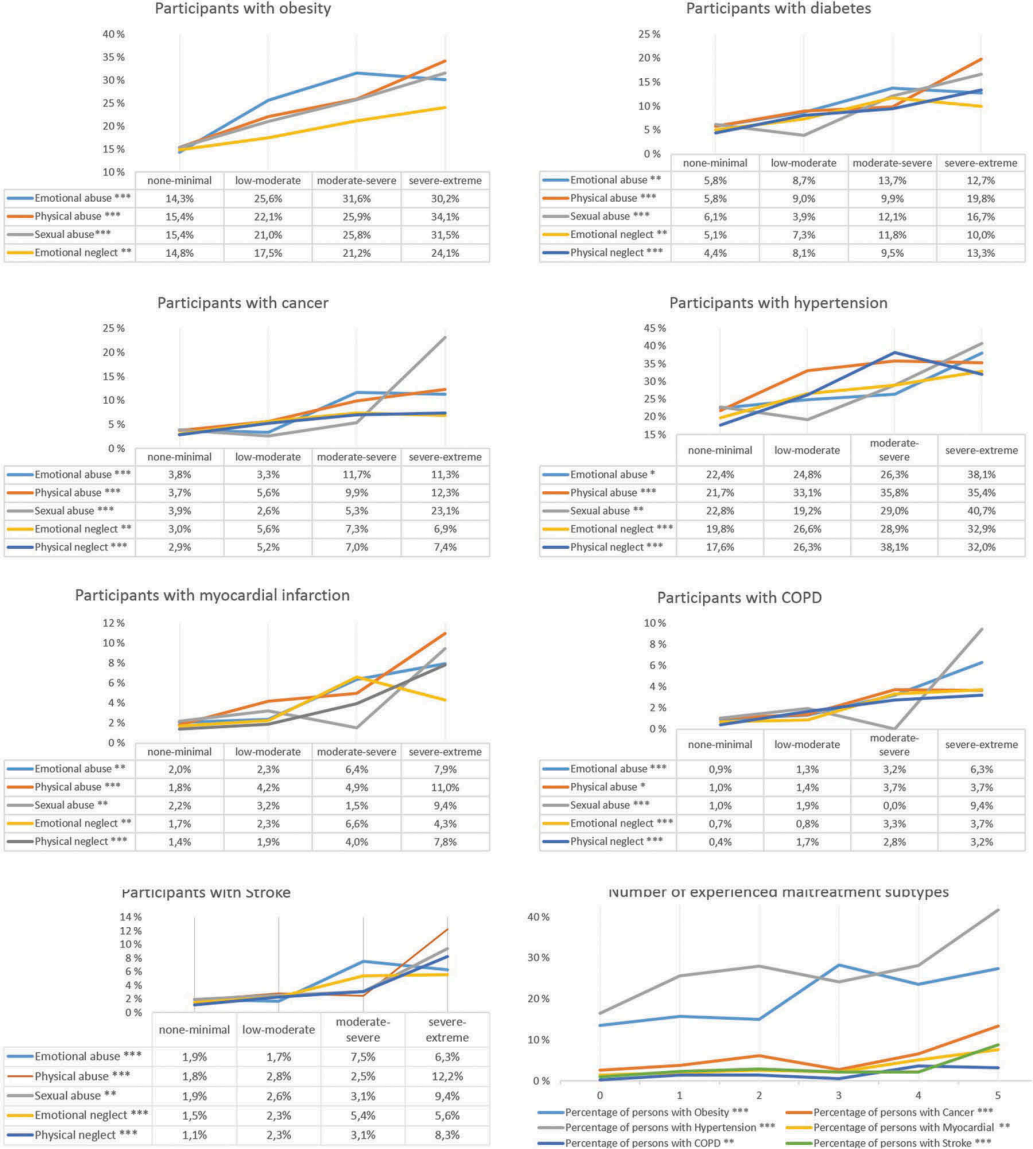
Health conditions in adulthood in association with different subtypes and levels of experienced child maltreatment. COPD, chronic obstructive pulmonary disease. ***p < 0.001, **p < 0.01, *p < 0.05.

The rates of all assessed health conditions increased when enhanced intensity of different subtypes of child maltreatment was reported. Furthermore, rates of all assessed health outcomes increased significantly with the rising number of subtypes of maltreatment experienced during childhood. Associations between increasing levels of different types of child maltreatment and different physical health conditions can be seen in Figure 1.

Having experienced any form of maltreatment was significantly associated with an increased risk for all physical health conditions (OR 1.45–5.29) (Table 2). In general, emotional and physical abuse, as well as emotional and physical neglect, were significantly associated with all health conditions (for physical neglect with the exception of obesity), while sexual abuse was only significantly associated with obesity, diabetes, cancer, COPD and stroke (Table 2).

**Table 2.** Odds ratios (OR) for health conditions in adulthood from participants with different subtypes and of the experience of any form of child maltreatment.

	Factor	Chi^2^	OR
Obesity	Emotional abuse ***	46.31	2.27
	Physical abuse ***	22.97	1.96
	Sexual abuse ***	17.54	1.78
	Emotional neglect **	7.88	1.36
	Physical neglect	2.07	1.17
	Any form of child maltreatment ***	10.26	1.45
Diabetes	Emotional abuse ***	12.29	1.87
	Physical abuse ***	16.96	2.22
	Sexual abuse *	4.17	1.53
	Emotional neglect **	10.94	1.72
	Physical neglect ***	26.39	2.30
	Any form of child maltreatment ***	17.90	2.22
Cancer	Emotional abuse *	4.92	1.65
	Physical abuse ***	15.06	2.42
	Sexual abuse *	5.89	1.79
	Emotional neglect ***	14.22	2.13
	Physical neglect ***	16.40	2.23
	Any form of child maltreatment **	10.55	2.10
Hypertension	Emotional abuse *	4.35	1.28
	Physical abuse ***	24.21	1.89
	Sexual abuse	2.16	1.22
	Emotional neglect ***	21.92	1.56
	Physical neglect ***	62.65	2.13
	Any form of child maltreatment ***	39.81	1.93
Myocardial infarction	Emotional abuse *	5.79	1.97
	Physical abuse ***	21.73	3.50
	Sexual abuse	2.21	1.62
	Emotional neglect *	6.10	1.93
	Physical neglect ***	15.06	2.82
	Any form of child maltreatment *	5.46	2.04
COPD	Emotional abuse *	6.62	2.58
	Physical abuse *	5.67	2.61
	Sexual abuse *	4.24	2.30
	Emotional neglect *	4.99	2.39
	Physical neglect ***	18.51	5.74
	Any form of child maltreatment **	9.20	5.29
Stroke	Emotional abuse *	4.48	1.88
	Physical abuse ***	14.39	3.00
	Sexual abuse *	4.91	2.02
	Emotional neglect **	8.55	2.24
	Physical neglect ***	20.23	3.55
	Any form of child maltreatment **	9.28	2.82

COPD, chronic obstructive pulmonary disease.

***p < 0.001, **p < 0.01, *p < 0.05.

**Table 3.** Association of health conditions in adults and child maltreatment via binary regression analysis.

Condition	Factor	Wald	Exp (*B*) (95% CI)
Obesity (*n* = 2416)			
*r* = 0.04	Male gender ***	20.25	0.59 (0.47–0.75)
	Age *	4.88	1.01 (1.00–1.01)
	Smoking	2.00	1.19 (0.94–1.50)
	Risk of or present alcohol abuse *	5.70	1.48 (1.07–2.04)
	High educational level (baccalaureate)	1.13	1.16 (0.89–1.51)
	Number of experienced maltreatment types ***	18.86	1.18 (1.10–1.27)
	Intercept ***	118.18	0.12
Diabetes (*n* = 2411)	Male gender	0.21	1.09 (0.77–1.53)
*r* = 0.15	Age ***	81.43	1.05 (1.04–1.06)
	Smoking	2.32	0.73 (0.48–1.10)
	Risk of or present alcohol abuse	0.87	1.27 (0.77–2.12)
	High educational level (baccalaureate)	0.09	1.07 (0.68–1.69)
	Number of experienced maltreatment types ***	15.42	1.26 (1.12–1.41)
	Intercept ***	216.21	0.00
Cancer (*n* = 2411)	Male gender	0.84	0.82 (0.54–1.25)
*r* = 0.12	Age ***	55.83	1.05 (1.04–1.07)
	Smoking	2.51	1.45 (0.92–2.30)
	Risk of or present alcohol abuse	0.12	1.12 (0.60–2.09)
	High educational level (baccalaureate)	0.65	1.25 (0.73–2.15)
	Number of experienced maltreatment types ***	12.33	1.28 (1.11–1.46)
	Intercept ***	170.13	0.00
Hypertension (*n* = 2414)	Male gender	0.06	0.97 (0.79–1.21)
*r* = 0.24	Age ***	276.75	1.06 (1.05–1.07)
	Smoking	0.16	0.95 (0.75–1.20)
	Risk of or present alcohol abuse	3.75	1.37 (1.00–1.15)
	High educational level (baccalaureate)	0.93	0.87 (0.66–1.15)
	Number of experienced maltreatment types ***	15.66	1.16 (1.08–1.25)
	Intercept ***	378.31	0.01
Myocardial infarction (*n* = 2410)	Male gender ***	18.42	4.07 (2.14–7.72)
*r* = 0.20	Age ***	42.12	1.07 (1.05–1.09)
	Smoking	0.42	0.81 (0.42–1.55)
	Risk of or present alcohol abuse	0.40	1.28 (0.60–2.76)
	High educational level (baccalaureate)	1.95	0.51 (0.20–1.32)
	Number of experienced maltreatment types **	7.42	1.29 (1.07–1.55)
	Intercept ***	129.32	0.00
COPD (*n* = 2411)	Male gender	0.04	0.92 (0.41–2.05)
*r* = 0.11	Age ***	13.55	1.05 (1.02–1.07)
	Smoking	1.14	1.60 (0.67–3.81)
	Risk of or present alcohol abuse	2.18	0.22 (0.03–1.66))
	High educational level (baccalaureate)	0.71	1.55 (0.56–4.33)
	Number of experienced maltreatment types **	11.18	1.51 (1.19–1.93)
	Intercept ***	70.86	0.00
Stroke (*n* = 2397)	Male gender	0.2	1.14 (0.64–2.06)
*r* = 0.15	Age ***	39.58	1.07 (1.05–1.09)
	Smoking	0.75	1.12 (0.57–2.20)
	Risk of or present alcohol abuse	0.10	1.14 (0.48–2.73)
	High educational level (baccalaureate)	0.18	0.83 (0.34–2.00)
	Number of experienced maltreatment types **	8.33	1.31 (1.09–1.58)
	Intercept ***	112.41	0.00

COPD, chronic obstructive pulmonary disease.

***p < 0.001, **p < 0.01, *p < 0.05.

### 3.2. Association of health conditions in adulthood and number of experienced types of child maltreatment

Binary regression analyses revealed significantly enhanced risks for all analysed health outcomes with increasing numbers of types of child maltreatment, when controlled for gender, age, smoking and risk for alcohol abuse (Table 3).

## 4. Discussion

This study was the first to investigate the association of child maltreatment with health conditions later in life in a representative sample of the German population. The present analyses show increased risks for obesity, cancer, hypertension, myocardial infarction, COPD and stroke if any kind of maltreatment had occurred during childhood. Furthermore, all health conditions were positively associated with greater intensity of maltreatment for each subtype as well as with increasing number of experienced maltreatment subtypes. Regression analysis revealed enhanced risks for all health conditions with increasing number of experienced subtypes of child maltreatment after controlling for gender, age, smoking and alcohol abuse. These results underline a cumulative association of child maltreatment types with various types of physical health conditions later in life.

Even though there has been a number of studies investigating the association of child maltreatment and physical health in adulthood, there is a lack of analyses assessing the relevance of physical, emotional and sexual abuse, as well as physical and emotional neglect, in one data set, while controlling for factors such as smoking and alcohol abuse.

Regarding the existence of associations between maltreatment during childhood and long-term physical health, the data presented herein are consistent with previous reports. For example, the ACE study, which included 9508 participants, found increased ORs in association with the number of maltreatment types experienced for the health conditions assessed in this study (Felitti et al., 1998). Similarly, another cross-sectional survey, which included data on 53,998 individuals from the USA, found increased ORs for diabetes, myocardial infarction and stroke depending on the number of adverse childhood events reported (Gilbert et al., 2015). The same holds true for a cross-sectional study with an Australian sample of more than 21,000 participants, showing increased ORs for stroke or myocardial infarction and emphysema if a person had experienced either physical or sexual abuse during childhood, while the odds increased further when both sexual and physical abuse had been experienced (Draper et al., 2008). This cumulative association was also shown for COPD and diabetes, where the odds were highest when both child physical and sexual abuse had been experienced (Shields, Hovdestad, Gilbert et al., 2016; Shields, Hovdestad, Pelletier et al., 2016). Our data support the potential cumulative association between child maltreatment and the development of health conditions later in life.

Regarding the association of each individual subtype with health conditions in adulthood, significantly increased rates were seen for nearly all analyses, especially for physical and emotional neglect, which have been analysed far less in previous studies. Most importantly, the ORs are comparable with the size of the odds for the three forms of abuse. This result is consistent with data from an analyses showing a comparable size of increase in the odds for hypertension if physical or sexual abuse or physical neglect occurred (Suglia et al., 2014). Especially with regard to the high prevalence rates of emotional and physical neglect, which are two- to three-fold higher than rates for abuse (Schilling et al., 2016; Witt et al., 2017), this points towards a potentially underestimated risk factor for many common, life-threatening diseases.

The pivotal role for sexual abuse, which was seen for the association between COPD and diabetes in the analyses by Shields and colleagues (Shields, Hovdestad, Gilbert et al., 2016; Shields, Hovdestad, Pelletier et al., 2016), was not confirmed in the present data. Nevertheless, in their analyses regarding COPD, results were presented stratified for gender, with markedly higher ORs for women compared to men, for whom no significant association was demonstrated (Shields, Hovdestad, Gilbert et al., 2016). The same difference in gender was seen for hypertension in the analyses by Suglia et al. (2014). As the present data combine results for men and women, this may explain the discrepant results. Regarding the association between diabetes and sexual abuse, the ORs presented here are comparable in size to those in the analyses of Shields, Hovdestad, Pelletier et al. (2016).

Several potential pathways could mediate the link between child maltreatment and health conditions in adulthood. One underlying biological mechanism, a dysregulation of the hypothalamic–pituitary–adrenal (HPA) axis, a major stress response in the human body, is discussed. Long-term alterations in the regulation of the HPA axis and its final product, cortisol, are a known consequence of the enormous stress caused by maltreatment during childhood (Carpenter et al., 2007), and are linked to health outcomes such as cancer and cardiovascular and metabolic diseases (Kumari, Shipley, Stafford, & Kivimaki, 2011; Volden & Conzen, 2013). Furthermore, chronic inflammatory processes, encompassing increased levels of pro-inflammatory cytokines and oxidative stress, are a demonstrated long-term consequence of child maltreatment (Boeck et al., 2016) and are known to play a role in the pathomechanisms of cancer (Taniguchi & Karin, 2018), cardiovascular diseases (Harrington, 2017), diabetes and other metabolic diseases (Herder et al., 2016). As well as biological mechanisms, socioeconomic and behavioural aspects are considered to be relevant for the demonstrated link between child maltreatment and health conditions in adulthood. The discussed pathways comprise altered health behaviour, encompassing a higher risk for substance abuse and risky sexual behaviour (Abajobir, Kisely, Williams, Strathearn, & Najman, 2017; Jewkes, Dunkle, Nduna, Jama, & Puren, 2010; Norman et al., 2012) and higher rates of smoking (Edwards, Anda, Gu, Dube, & Felitti, 2007; Taha, Galea, Hien, & Goodwin, 2014), as well as strikingly increased rates of mental health problems and suicide attempts (Lereya, Copeland, Costello, & Wolke, 2015; Norman et al., 2012; Winsper, Lereya, Zanarini, & Wolke, 2012). Hypothesized socioeconomic aspects comprise impaired social networks and relationships (Colman & Widom, 2004; Sperry & Widom, 2013), lower academic achievement (Fry et al., 2017; Tanaka, Georgiades, Boyle, & MacMillan, 2015) and lower socioeconomic status (Currie & Widom, 2010; Pinto Pereira, Li, & Power, 2017; Zielinski, 2009).

### 4.1. Limitations

Analyses assessing individual maltreatment subtypes were performed with the chi-squared test. No controlling for age was carried out. The risk for all of the assessed health conditions is known to increase with age and, furthermore, the prevalence of neglect, but also abuse, is known to be higher in older age groups, as was shown in another study on the sample used herein (Witt et al., 2017). Therefore, the results of the chi-squared analyses can be interpreted only with care. Nevertheless, the results of the regression analysis, which controlled for age, confirm the results regarding the association between health conditions in adulthood and the number of subtypes of child maltreatment that were experienced.

One important limitation of this study is that the results are based on a retrospective self-report, potentially involving recall bias. In particular, the risk of underreporting due to recall bias, denial, embarrassment and misunderstanding may affect the results (Fergusson, Horwood, & Woodward, 2000; R. Gilbert et al., 2009). Nevertheless, in the field of child maltreatment, which is characterized by significant underreporting, as, for example, only 5–8% of abuses lead to contact with child protection services, resulting in severe selection bias (MacMillan, Jamieson, & Walsh, 2003), anonymous retrospective self-ratings are a highly valuable source of information.

As this is an observational study, causality cannot be deduced. Nevertheless, the data on the presented analysis give a meaningful insight into the relevance of child maltreatment for the analysed health conditions in adulthood, especially as they are comparable to international analyses that include longitudinal data.

### 4.2. Conclusions

Child maltreatment is cumulatively associated with increased risks for the leading causes of morbidity and mortality in Germany (DESTATIS Statistisches Bundesamt). Child maltreatment may need to be understood as a still underestimated risk factor for the development of these widely devastating diseases, resulting in fatal consequences on an individual as well as a socioeconomic level. Interventions encompassing both secondary preventive strategies, through education about the increased risk for the development of these diseases, and primary preventive strategies (such as training in parenting skills and supervision) are critical to address and target this major public health problem and its devastating consequences.
